# Emergence of an erythroid cell-specific regulatory region in *ABO* intron 1 attributable to A- or B-antigen expression on erythrocytes in Hominoidea

**DOI:** 10.1038/s41598-023-31961-6

**Published:** 2023-03-27

**Authors:** Rie Sano, Haruki Fukuda, Rieko Kubo, Takao Oishi, Takako Miyabe-Nishiwaki, Akihisa Kaneko, Haruhisa Masato, Yoichiro Takahashi, Akira Hayakawa, Shin Yazawa, Yoshihiko Kominato

**Affiliations:** 1 Department of Legal Medicine, Gunma University Graduate School of Medicine, 3-39-22 Showa-Machi, Maebashi, 371-8511 Japan; 2 Center for the Evolutionary Origins of Human Behavior, Kyoto University, Inuyama, Japan; 3 Hitachi City Kamine Zoo, Hitachi, Japan; 4 Department of Legal Medicine, Faculty of Medicine, University of Tsukuba, Tsukuba, Japan; 5 Department of General Surgical Science, Graduate School of Medicine, Gunma University, Maebashi, Japan

**Keywords:** Evolutionary biology, Gene expression, Gene regulation, Genome, DNA

## Abstract

A- and B-antigens are present on red blood cells (RBCs) as well as other cells and secretions in Hominoidea including humans and apes such as chimpanzees and gibbons, whereas expression of these antigens on RBCs is subtle in monkeys such as Japanese macaques. Previous studies have indicated that H-antigen expression has not completely developed on RBCs in monkeys. Such antigen expression requires the presence of H-antigen and A- or B-transferase expression in cells of erythroid lineage, although whether or not ABO gene regulation is associated with the difference of A- or B-antigen expression between Hominoidea and monkeys has not been examined. Since it has been suggested that ABO expression on human erythrocytes is dependent upon an erythroid cell-specific regulatory region or the + 5.8-kb site in intron 1, we compared the sequences of *ABO* intron 1 among non-human primates, and demonstrated the presence of sites orthologous to the + 5.8-kb site in chimpanzees and gibbons, and their absence in Japanese macaques. In addition, luciferase assays revealed that the former orthologues enhanced promoter activity, whereas the corresponding site in the latter did not. These results suggested that the A- or B-antigens on RBCs might be ascribed to emergence of the + 5.8-kb site or the corresponding regions in *ABO* through genetic evolution.

## Introduction

The ABO blood group system was discovered by Karl Landsteiner in 1900^1,2^. Histo-blood group ABH(O) antigens are characterized as defined trisaccharide determinants GalNAc α1 → 3 [Fucα1 → 2]Galβ1 → R, Galα1 → 3[Fucα1 → 2]Galβ1 → R, and disaccharide determinants Fucα1 → 2Gal β1 → R for A, B, and H, respectively^3,4^. Gal β1 → R denotes core chains such as Galβ1 → 3GlcNAc and Galβ1 → 4GlcNAc. These antigens are present on red blood cells (RBCs), in secretions, and on epithelial cells and vascular endothelial cells^5–9^. Their structures are synthesized from the precursor H substrate by α1 → 3GalNAc transferase (A-transferase) and α1 → 3Gal transferase (B-transferase), encoded by functional alleles at the ABO locus^10,11^. On the other hand, the precursor H substrate on RBCs is produced by α1 → 2Fuc transferase (H-transferase) encoded by *FUT1*^4^. ABH-antigens are present at the non-reducing ends of carbohydrate chains, which are produced by sequential transfer of sugar by each glycosyltransferase specific to the non-reducing end of a precursor or immature carbohydrate chain. Thus, orchestrated expression of associated glycosyltransferases is needed for production of a carbohydrate chain, although the underlying mechanism responsible for regulation of the genes encoding the glycosyltransferases involved in carbohydrate chain production remains unclear.

**Figure 1:**
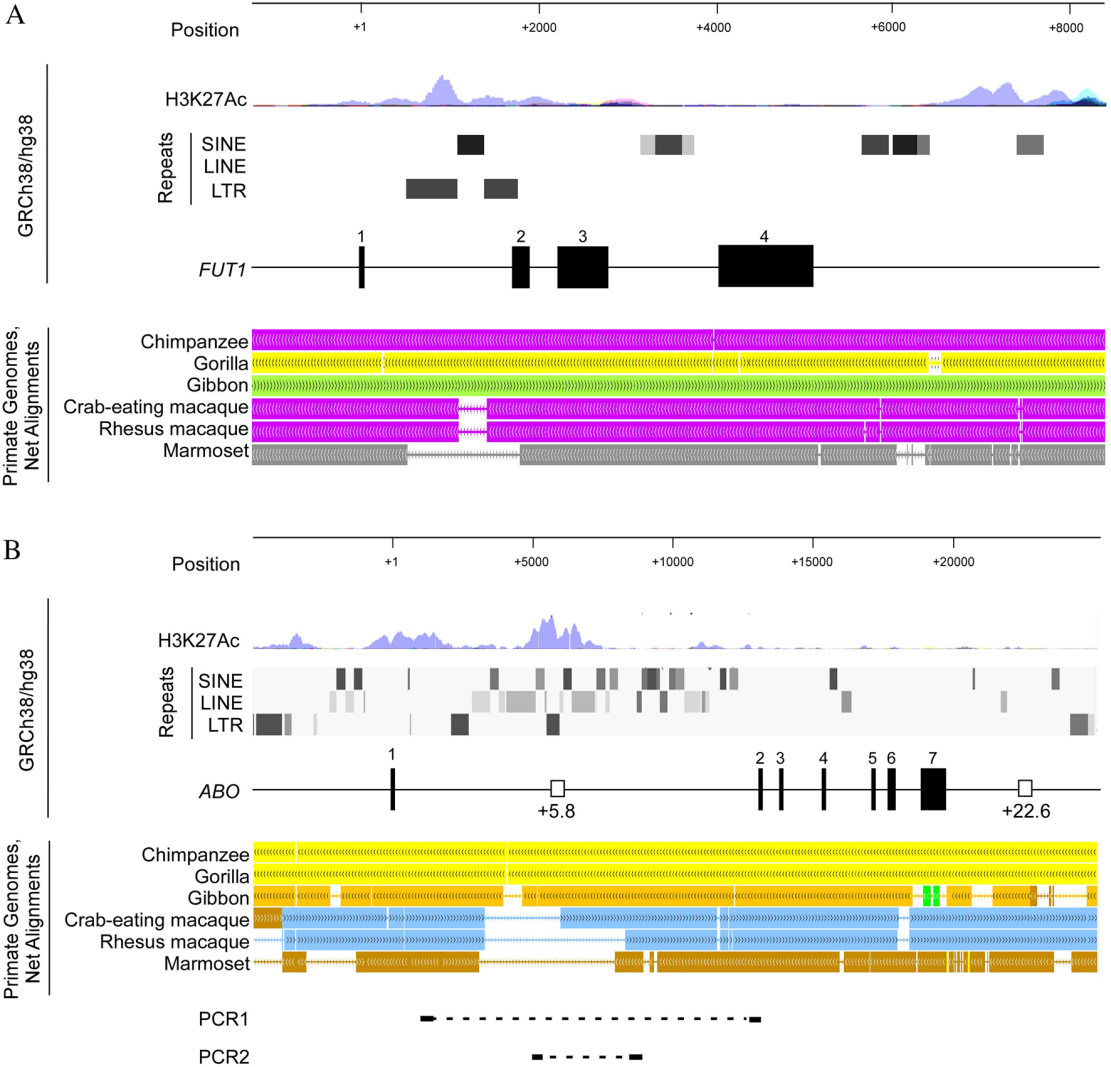
Homology of nucleotide sequences from the upstream to downstream regions of *FUT1* or *ABO* between human and non-human primates. (**A**) Human *FUT1*, (**B**) Human *ABO*. Upper panel shows positions relative to the transcription start site of *FUT1* or *ABO*. The second panel from the top indicates the acetylation at lysine 27 of histone 3 in *FUT1* or *ABO*, often found near active regulatory elements, which was demonstrated by ENCODE Regulation Tracks. The third panel from the top denotes repeating elements including SINE, LINE, and LTR, which were revealed by RepeatMasker over the genomic structure of *FUT1* or *ABO*. The fourth panel shows the genomic structure from the upstream region through *FUT1* or *ABO* to the downstream region, including exons, as well as regulatory regions such as the + 5.8- and + 22.6-sites for erythroid cells and epithelial cells, respectively, on the basis of the human genome draft GRCh38/hg38. Exons are denoted by filled boxes, and the regulatory regions are indicated by empty boxes. The fifth panel shows a comparison of the genome sequences for human with their reference sequences for non-human primates including chimpanzee, gorilla, gibbon, crab-eating macaque, rhesus macaque, and marmoset using Primate Genomes, Chain and Net Alignments^18^. Nucleotides with high identity are highlighted. The bottom panel of (**B**) shows the locations of PCR-amplified fragments of PCR1 and PCR2.

ABO blood groups are determined by these antigens on RBCs, as well as anti-A and anti-B antibodies in serum in accordance with Landsteiner’s law. Weak expression of the ABH-antigen on RBCs is associated with ABO subgroups or variant types^12^. Similar reduced reactivity of RBCs with anti-A or anti-B antibody is observed in non-human primates. Studies using routine anti-A and anti-B reagents have shown that agglutination of RBCs in apes such as chimpanzees, gorillas, orangutans, and gibbons is similar to that of human subgroups such as A_int_ or B_int_^13,14^. A_int_ erythrocytes have lower amounts of A-antigen than A_1_ erythrocytes and higher amounts than A_2_ erythrocytes. The B_int_ is comparable to A_int_^15,16^. On the other hand, similar tests have shown that the RBCs of prosimians, New World monkeys and Old World monkeys appear to lack A- or B-antigens, although trace amounts have been demonstrated using very sensitive absorption-elution techniques or very potent anti-A or anti-B reagents^13,14,17^. Nakajima et al. have concluded that expression of H-antigen on RBCs has not completely developed in monkeys, because eluates from such RBCs obtained by absorption-elution tests with anti-H reagent were found to weakly agglutinate group O-RBCs^14^. One suggested potential mechanism leading to such increased expression of H-antigen on RBCs of human is insertion of a short interspersed nuclear element (SINE), one of several transposable elements, in the first intron of *FUT1* encoding H-transferase (Fig. 1A), since this element is present in Hominoidea including humans and apes, whereas it is absent in monkeys or non-primate mammals, which lack ABH-antigens on RBCs (Fig. 1A)^18–21^. Either A- or B-antigen expression on RBCs requires the presence of H-antigen as well as expression of A- or B-transferase in erythroid lineage cells. However, whether or not ABO gene regulation is associated with this difference of A- or B-antigens on RBCs between Hominoidea and monkeys has not been examined.

Yamamoto et al. have demonstrated that human *ABO* is composed of seven exons spanning 20 kb^22^. Subsequently, a positive regulatory element, named the + 5.8-kb site, in intron 1 was implicated in erythroid cell-specific regulation of *ABO* transcription as a result of in vitro experiments and genetic analysis of individuals with B_m_ harboring deletion of 5.8 kb or 3.0 kb including the site (Fig. 1B)^23,24^. As the site includes one transcription factor recognition motif for RUNX1 and two motifs for GATA1/2^25,26^, deletion and single-nucleotide substitutions in the RUNX motif in the + 5.8-kb site have been found in individuals with A_m_, B_3_ or B_w_^26–28^, while nucleotide substitutions in the downstream GATA motif of the site have been reported in individuals with B_m_ and A_m_^25,29,30^. Thus, erythroid cell-specific regulation of *ABO* transcription could be dependent upon the + 5.8-kb site and the constitutive *ABO* promoter^23,31,32^. In addition, in vitro experiments have shown that a positive regulatory element, named the + 22.6-kb site, downstream of *ABO* is associated with epithelial cell-specific regulation of *ABO* transcription (Fig. 1B)^33^. On the other hand, it remained to further investigate whether the *ABO* expression was dependent upon the + 5.8-kb site or the + 22.6-kb site in the vascular endothelium. However, given that hematopoietic stem cells and vascular endothelial cells are thought to have a common ancestral cell, the + 5.8-kb site might be involved in *ABO* transcriptional regulation in the vascular endothelium.

Here, we compared the sequences of the + 5.8-kb site in human *ABO* with those of the corresponding sites in primate species including chimpanzees, agile gibbons, and Japanese macaques, and carried out transient transfection experiments using reporter plasmids containing those sites. The results suggested that the appearance of A- and B-antigens on RBCs might be ascribable to emergence of the + 5.8-kb site or its orthologues through genomic evolution.

## Materials and methods

### Blood and saliva

Blood and saliva specimens were obtained at health check from several representative individuals of chimpanzee (*Pan troglodytes*), agile gibbon (*Hylobates agilis*), and Japanese macaque (*Macaca fuscata*) at the Center for the Evolutionary Origins of Human Behavior, Kyoto University. As mentioned briefly, the individual primates were anesthetized for a regular health examination. Anesthesia for chimpanzee was induced with intramuscular administration of the combination of medetomidine, midazolam, and ketamine and maintained with intravenous infusion of propofol^34^. Anesthesia for agile gibbon and Japanese macaque was induced with intramuscular administration of the combination of medetomidine, midazolam, and ketamine. Two or three ml of blood samples were obtained from the femoral vein and saliva were collected with a cotton swab. Experimental designs and procedures were approved under permit No.2021-037, by the Animal Welfare and Animal Care Committee at the Primate Research Institute, Kyoto University and all animal experiments were carried out in accordance with institutional guidelines for the care and use of nonhuman primates (http://www.pri.kyotou.ac.jp/research/sisin2010/Guidelines_for_Care_and_Use_of_Nonhuman_Primates20100609.pdf). All methods are reported in accordance with ARRIVE guidelines (https://arriveguidelines.org).

ABO blood groups of primates were determined using forward and reverse grouping of blood, and agglutination inhibition tests for ABH substances in saliva^14,35^. Briefly, for the forward grouping, one drop of 2% blood suspension was mixed with one drop of anti-A and anti-B antibodies (Wako) as well as *Ulex europaeus* lectin (J-Chemical) on a hole glass, and the agglutination reaction was observed 30 min after agitation at room temperature. For the reverse grouping, the plasma was mixed with human O blood cells overnight in a refrigerator to remove heterologous antibodies, and one drop each of the treated plasma and human A_1_ or B blood cells was agitated on a hole glass, followed by observation of the agglutination reaction. For agglutination inhibition tests for ABH substances in saliva, a cotton swab wiped with fresh saliva was moistened with 300 µl of saline solution and heated to denature the proteins. Equal amount of anti-A, anti-B or anti-H reagents with an eightfold aggregation titer were mixed with the diluted saliva after heat treatment for two hours at room temperature. Human A_1_, B, and O blood cells were then added to the saliva-antibody mixture of the corresponding types, respectively, and inhibition of hemagglutination by the blood group substance in the saliva was observed.

Genomic DNA was extracted from blood using a QIAamp blood mini kit (Qiagen GmbH).

### PCR amplification and cycle sequencing

PCR1 and PCR2 were carried out for PCR amplification of a portion of the first *ABO* intron in primate species (Fig. 1B). PCR1 used the primers hABOg997F and hABOex2R, whose sequences were 5′-AGAGGAGTGGAAAATTCATGAAGA-3′ and 5′-CCAAACAAGACCAAGACAAGCAT-3′, respectively. PCR amplification was performed in 50 μl of reaction mixture containing 200 ng of genomic DNA, 1 × KOD One PCR Master Mix (TOYOBO) with KOD DNA polymerase, and each primer at 0.1 μM. Amplification was carried out under the following conditions: initial denaturation at 98 °C for 3 min followed by 35 cycles of 98 °C for 10 s, 60 °C for 5 s and 68 °C for 10 min. PCR1 was followed by nested PCR2. PCR2 used the primers hABOc.28 + 5005 and hABOc.28 + 8800, whose sequences were 5′-TCGGCTCTTGCCAGGTGGAG-3′ and 5′-CCACAATATCTCAGGGACCCCATA-3′, respectively. PCR2 amplification was performed using the same reaction mixture as that for PCR1. Amplification was carried out under the following conditions: initial denaturation at 98 °C for 3 min followed by 35 cycles of 98 °C for 10 s and 68 °C for 2 min, followed by direct sequencing of PCR2 products with specific primers for the PCR2 target and the BigDye™ terminator kit version 1.1 (Thermo Fisher Scientific, Waltham, MA). The sequencing run was performed on a genetic analyzer (Thermo Fisher Scientific, Seqstudio). Also, the PCR2 products were ligated into the pUC 118 vector using a Mighty Cloning Reagent set (Blunt End) (TaKaRa), followed by determination of the nucleotide sequences of the clones obtained using the primers M13M4 and M13RV.

### Plasmids

Luciferase reporter plasmids SN, C(01)/SN, C(04)/SN, C(06)/SN, C(B_m_)/SN, have been described previously^25,26^. In reporter plasmid SN, the *ABO* proximal promoter located between ‒ 150 and ‒2 relative to the translation start site was subcloned upstream of the luciferase gene^23^, whereas the + 5.8-kb site between c.28 + 5624 and c.28 + 6125 with haplotype ABOInt1*01, ABOInt1*04, or ABOInt1*06 was subcloned upstream of the *ABO* promoter in reporter plasmid C(01)/SN, C(04)/SN, or C(06)/SN, respectively^25^. The regions in the first intron 1 of *ABO* in non-human primates that corresponded to the human + 5.8-kb site were generated by PCR using specific primers and the PCR2 products as templates, followed by insertion just upstream of the human *ABO* promoter in constructs C(Chi)/SN, C(Gib)/SN, and C(Mac)/SN. The directions of the inserts of all constructs used in this study were verified by DNA sequence analysis as described above. Plasmid DNA was purified using a plasmid purification kit (HiSpeed Plasmid Maxi kit, Qiagen GmbH).

### Transfection and luciferase assay

Transient transfection into K562 cells was performed using Lipofectamine 3000 (Invitrogen Corp.) as reported previously^23^. For each analysis, 2.5 μg of firefly luciferase reporter plasmid and 0.01 μg of pRL-SV40 *Renilla* luciferase reporter vector were used. After the cells had been collected, cell lysis and luciferase assays were performed using the dual-luciferase reporter assay system (Promega) to measure the activities of the firefly and *Renilla* luciferases. Light emission was measured using an absorption spectrophotometer (Nivo F, Perkin Elmer). Variations in transfection efficiency were normalized to the activities of *Renilla* luciferase expressed from the cotransfected pRL-SV40 *Renilla* luciferase reporter.

## Results

### Comparison of genomic DNAs from the upstream to downstream region of *ABO* among humans and primate species

Primate Genomes, Chain and Net Alignments^18^ from the UCSC Genome Browser for humans and primate species have demonstrated high sequence conservation between the ATG translation start codon and the stop codon of *ABO*, except for a few regions including the + 5.8-kb site (Fig. 1B). The + 5.8-kb site is conserved among human, chimpanzee, gorilla, and gibbon, showing similar expression of A- and B-antigens on RBCs^13^, whereas it is not conserved in monkeys such as crab-eating macaque, rhesus macaque and marmoset, in which the A- and B-antigens are expressed only slightly on RBCs. On the other hand, the + 22.6-kb site is conserved among humans and those primate species. Therefore, further study was carried out to examine whether the + 5.8-kb site could contribute to the presence or absence of A- and B-antigens on RBCs using blood and saliva specimens from several representative individuals of chimpanzee, agile gibbon, and Japanese macaque without any sanguineous relationship.

### Serological tests and phenotypes

Serological tests were performed for two individual chimpanzees (Table 1). Their RBCs were agglutinated by anti-A antibody, but not by anti-B antibody. Their sera contained antibodies that agglutinated B-RBCs, and lacked antibodies that agglutinated A-RBCs. Consistently, their saliva was shown to contain A- and H-substances by saliva tests, based on its ability to inhibit anti-A and anti-B reagents. Thus, their blood types were determined as A. Serological tests were also carried out for three individual agile gibbons (Table 1). The RBCs from two individuals were agglutinated by anti-A and anti-H antibodies, while RBCs from the other individual were agglutinated by anti-B antibody, but not by anti-A antibody. The sera from the former individuals lacked antibodies that agglutinated A- and B-RBCs, while the serum from the latter individual contained antibody that agglutinated A-RBCs and lacked antibodies that agglutinated B-RBCs. Consistently, the saliva from the two individuals was shown to contain A-, B- and H-substances, while that from the other contained B- and H-substances. Thus, the blood type of the former individuals was determined as AB, whereas that of the latter individual was determined as B. When serological tests were carried out on three individual Japanese macaques, their RBCs were not agglutinated with anti-A and anti-B antibodies (Table 1). The serum from one individual contained antibody that agglutinated A-RBCs and lacked antibodies that agglutinated B-RBCs, while that from the others contained antibody that agglutinated both A-RBCs and B-RBCs. Consistently, the saliva from one individual was shown to contain B- and H-substances, while that from the others contained only H-substances. Thus, the blood type of the one individual was determined as B, whereas that of the other two individuals was determined as O. In conclusion, the presence of A- or B-antigens on RBCs was concordant with that in saliva in chimpanzees and agile gibbons. On the other hand, B-antigens were not detectable from RBCs of a Japanese macaque, but were found in saliva. This phenotype seemed to correspond to the weak human phenotype B_m_.

**Table 1:** Summary of serological results obtained using samples from individual primates.

Individual	Species	Blood				*ABH-antigen in saliva	ABO phenotype
		Agglutinability of red cells		Reaction of serum			
		Anti-A	Anti-B	Human A-RBC	Human B-RBC		
#1	Chimpanzee	^‡^4 +	−	−	+	H, A	A
#2	Chimpanzee	4 +	−	−	+	H, A	A
#3	Agile gibbon	4 +	4 +	−	−	H, A, B	AB
#4	Agile gibbon	4 +	4 +	−	−	H, A, B	AB
#5	Agile gibbon	−	4 +	+	−	H, B	B
#6	Japanese Macaque	−	−	+	+	H	O
#7	Japanese Macaque	−	−	+	−	H, B	B
#8	Japanese Macaque	−	−	+	+	H	O

*Presence of ABH-antigens was examined by testing saliva for its ability to inhibit anti-A, anti-B and anti-H reagents.

^‡^4 + denotes complete agglutination observed.

### LTRs at sites corresponding to the human + 5.8-kb site was preserved in chimpanzees and agile gibbons, and absent in Japanese macaques

**Figure 2:**
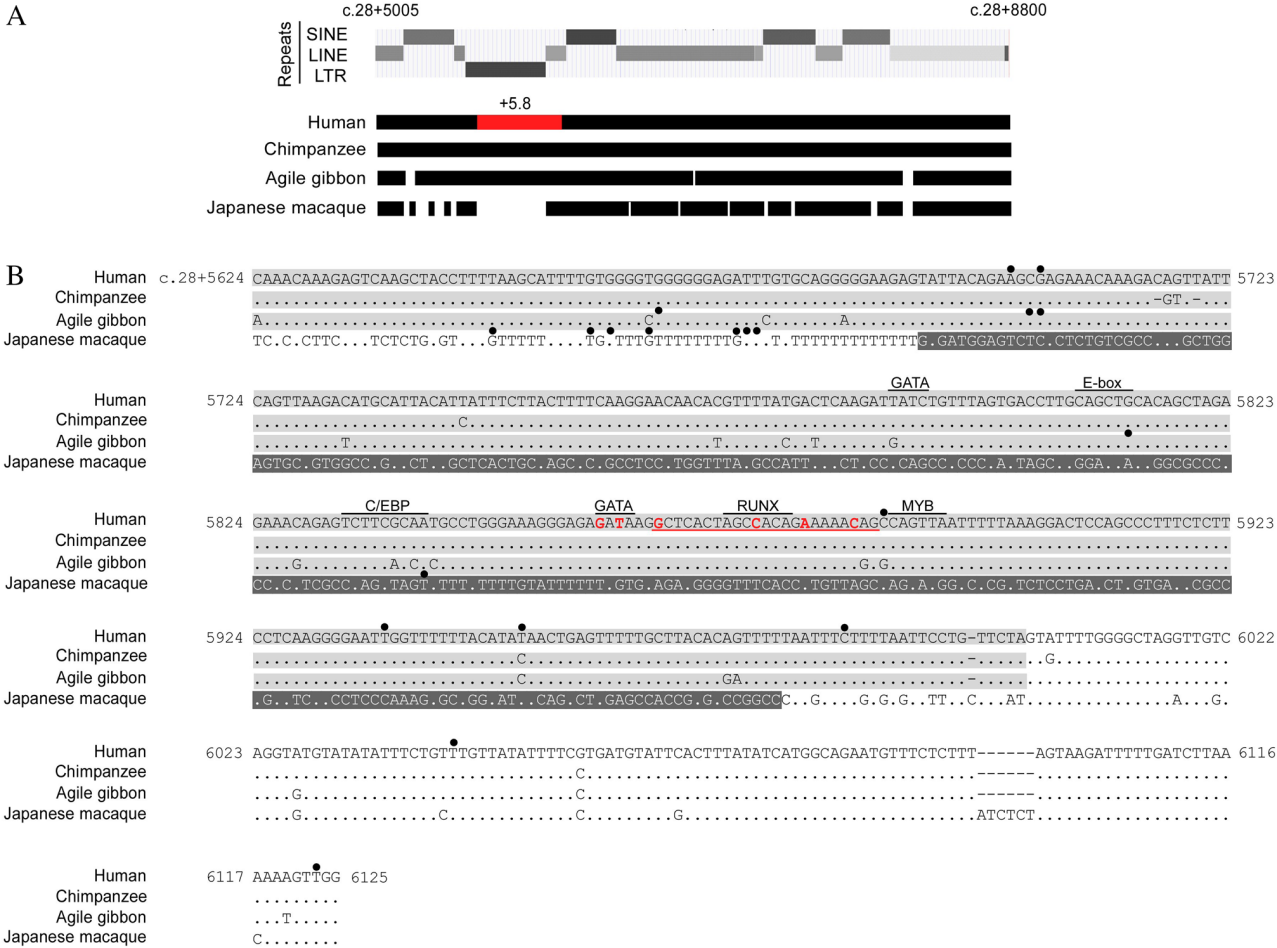
Comparison of nucleotide sequences in the first intron of *ABO* involving the human + 5.8-kb site and the corresponding regions in chimpanzee, gibbon, and Japanese macaque. (**A**) Comparison of nucleotide sequences in the first intron of *ABO* among human, chimpanzee, gibbon, and Japanese macaque. The top line shows the nucleotide sequence of the first intron in human *ABO* from c.28 + 5005 to c.28 + 8800, and the + 5.8-kb site in human *ABO* is denoted in red. The other lines represent the nucleotide sequences in chimpanzee, gibbon, and Japanese macaque corresponding to the human genome. Retrotransposons in the human genome are denoted over those lines for human and non-human primates. (**B**) Comparison of nucleotide sequences between the human + 5.8-kb site and the corresponding sites found in non-human primates. The top line shows the nucleotide sequence of the + 5.8-kb site in human *ABO* from c.28 + 5624 to c.28 + 6125, and the other lines represent the nucleotide sequences of the sites corresponding to the human + 5.8-kb site from chimpanzee, gibbon, and Japanese macaque. The sequences were deposited in GenBank with accession numbers OP775662 (chimpanzee), OP775663 (gibbon), and OP775664 (Japanese macaque). Nucleotides that are red or underlined indicate single nucleotide variants (SNVs) or deletion in individuals with weak phenotypes such as A_m_, A_3_, B_m_ and B_3_. The motifs for transcription factor recognition sites are indicated by overbars. For comparison with the nucleotides in human *ABO*, the substituted nucleotides are shown and horizontal bars denote deletion of nucleotides in those monkeys. Solid circles over nucleotides in the human and monkey sequences indicate single nucleotide polymorphisms (SNPs) or variants. Nucleotide sequences belonging to LTR are highlighted in gray, and those belonging to SINE are highlighted in dark gray.

Further investigation was carried out to clarify the mechanism responsible for loss or reduction in B-antigen expression in the above Japanese macaque. Nested PCR amplification was performed to determine the sequences of 3.8-kb in the first intron, which corresponded to the regions including the upstream sites corresponding to the human + 5.8-kb site to the downstream stretch using the genomic DNAs obtained from two individual chimpanzees, three individual agile gibbons, and three individual Japanese macaques. When the nucleotide sequences from c.28 + 5005 to c.28 + 8800 in the first intron of human *ABO* were compared with those among the chimpanzees, gibbons, and Japanese macaques, most of the chimpanzee and gibbon nucleotide sequences were similar to those of the human + 5.8-kb site, although in Japanese macaques several regions were not conserved (Fig. 2A). Specifically, regarding the + 5.8-kb site, the human nucleotide sequences from c.28 + 6002 to c.28 + 6125 were conserved among the four primates (Fig. 2B). In contrast, the human sequences from c.28 + 5624 to c.28 + 6001 were conserved in the chimpanzees and agile gibbons, but not in Japanese macaques. The human sequences from c.28 + 5624 to c.28 + 6001 were shown to belong to long terminal repeats (LTR) by RepeatMasker^36^, and the LTR was located between long interspersed nuclear elements (LINE) that were classified as L1MB7 (Fig. 2A). However, the site corresponding to the + 5.8-kb site included SINE in Japanese macaques (Fig. 2B). Therefore, since the + 5.8-kb site plays a role in erythroid cell-specific expression of human *ABO*, it seemed likely that the LTR of the human + 5.8-kb site and its orthologues could be involved in erythroid cell-specific regulation of *ABO* expression in human, chimpanzee and agile gibbon.

### Significance of other LTRs similar to the LTR in the + 5.8-kb site as a positive regulatory element in humans

To examine whether other LTRs similar to that in the + 5.8-kb site might play a role in gene regulation in humans, a BLAST search was performed using the query sequence of the LTR in the human + 5.8-kb site, as the LTR was classified group *HERVER1* according to Vargiu et al.^37^ or LTR15 by RepeatMasker. Above an identity of 92%, 30 LTRs are listed in Table 2, 19 of them belonging to LTR15. ENCODE Candidates Cis-regulatory Elements^38^ indicated 10 LTRs with a distal enhancer-like signature, a promoter-like signature or DNase-H3K4me3, although GeneHancer Regulatory Elements and Gene Interactions^39^ showed no interaction configuration in the LTRs other than that in the + 5.8-kb site which had been shown previously to interact with the transcription start sites of genes including *ABO*, *SURF6*, *MED22*, *RPL7A*, *SNORD24*, *SNORD36*, *SURF1*, *SURF2*, and *DBH* in humans^40^. Thus, it was unclear whether other LTRs similar to that in the + 5.8-kb site could be involved in gene expression in humans, and heterogeneity for positive regulatory potential of the LTRs might be dependent upon local genomic circumstances or positional effects.

**Table 2:** Sequences homologous to the human + 5.8-kb site demonstrated by a BLAST search with candidates for estimated cis-regulatory elements.

Position*	Identity %	Cis-regulatory elements^§^	Location	
chr9:133269164–133269541^†^	99.5	Distal enhancer-like signature	*ABO*	Intron 1
chr1:154291653–154292344^†^	94.2	Distal enhancer-like signature	Intergenic region	
chr14:94084570–94084951^†^	93.9	None	*IFI27L1*	Intron 1
chr3:145546392–145546770^†δ^	93.6	DNase-H3K4me3	Intergenic region	
chr10:25279470–25279845^†^	93.5	None	*GPR158*	Intron 3
chr19:19826275–19826655	93.3	None	*ZNF56P*	Intron 1
chr9:36304059–36304746^†^	93.3	None	Intergenic region	
chr6:150365509–150365886^†^	92.9	None	Intergenic region	
chr1:197097013–197097382^†^	92.9	None	*ASPM*	Intron 17 or 18
chr3:146922327–146922695	92.9	None	Intergenic region	
chr17:39334919–39335299^†^	92.8	Distal enhancer-like signature	*FBXL20*	Intron 2
chr12:86129189–86129890^†^	92.8	Distal enhancer-like signature	*MGAT4C*	Intron 1 or 4
chr13:57436874–57437242^†δ^	92.6	Distal enhancer-like signature	Intergenic region	
chr14:88819677–88820049^†^	92.5	None	ENSG00000274492	Exon 1
chr2:149240139–149240517^†^	92.4	None	Intergenic region	
chr16:3085288–3085666^†^	92.3	Promoter-like signature	ENSG00000262370	Intron 1
chr4:44032311–44032686	92.3	None	Intergenic region	
chr19:49124931–49125639	92.3	None	*SPHK2*	Intron 2
chr14:19776986–19777376	92.3	None	Intergenic region	
^†^chr4:156063554–156063924	92.2	Distal enhancer-like signature	Intergenic region	
chr4:156984706–156985076	92.2	None	Intergenic region	
chr11:24047586–24047954	92.2	None	Intergenic region	
chr8:522284–522666^†^	92.1	None	*TDRP*	Intron 1
chr16:31241396–31241760	92.1	None	Intergenic region	
chr4:99080845–99081222	92.0	None	*ADH5*	Intron 4
chr12:9392179–9392557^†^	92.0	None	*LINC02367*	Intron 2
chr1:67141852–67142227^†^	92.0	Distal enhancer-like signature	*C1orf141, IL23R*	Intron 1
chr19:44573691–44574071^†^	92.0	Distal enhancer-like signature	Intergenic region	
chr7:106630097–106630468	92.0	None	Intergenic region	
chr22:16065595–16065985	92.0	None	Intergenic region	

*Positions are described according to Human GRCh38/hg38. For the BLAST, a portion of the + 5.8-kb site of haplotype ABOInt1*01 belonging to LTR15 was used as the query sequence. Since the + 5.8-kb site in human GRCh38/hg38 belonged to haplotype ABOInt1*04, there was a difference of two nucleotides between these + 5.8-kb sites.

^‡^Descriptions are according to ENCODE Candidates Cis-regulatory Elements (cCREs).

^†^Sequences indicated to belong to LTR15 by RepeatMasker.

^δ^The regions chr3:145546392–145546770 and chr13:57436874–57437242 belonging to LTR15 were located between LINEs of L1M1 and L1PBa1, respectively. Similarly, the human + 5.8-kb site belonging to LTR15 was located between L1MB7s.

### Examination of the *trans*-activation potential of sites corresponding to the human + 5.8-kb site in chimpanzee, agile gibbon, and Japanese macaque

**Figure 3:**
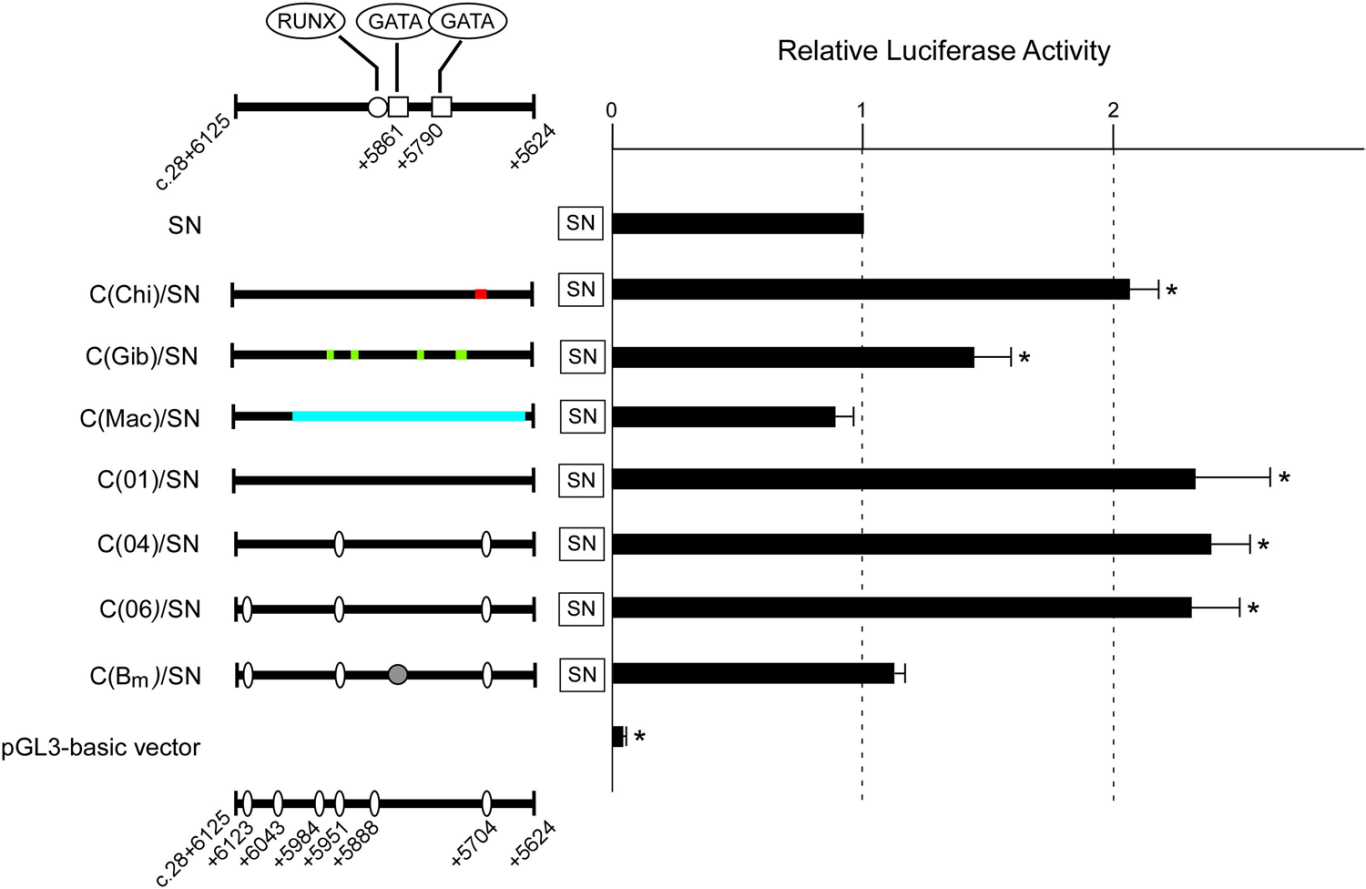
Transcriptional regulatory activity of the human + 5.8-kb site was lost by change from its homologs in apes to the corresponding site in Japanese macaque. K562 cells were transiently transfected with *ABO* enhancer-luciferase gene constructs including SN, C(01)/SN, C(04)/SN, C(06)/SN, and C(B_m_)/SN as well as C(Chi)/SN, C(Gib)/SN, and C(Mac)/SN. Reporter plasmid SN contained the human *ABO* promoter, and reporter plasmids C(01)/SN, C(04)/SN, and C(06) comprised the promoter and + 5.8-kb site of haplotypes ABOInt1*01, ABOInt1*04, and ABOInt1*06, respectively. Haplotypes ABOInt1*01, ABOInt1*04, and ABOInt1*06 were linked to the *O* allele, *A* allele and *B* allele, respectively, with a few exceptions attributable to genetic recombination^49^. Locations of the single nucleotide polymorphisms are shown by ovals. Reporter plasmid C(B_m_)/SN included the promoter and + 5.8-kb site harboring a mutation of the GATA recognition site in the background of haplotype ABOInt1*01^25^. Location of the mutation is denoted by a solid circle. Reporter plasmids C(Chi)/SN, C(Gib)/SN, or C(Mac)/SN contained the promoter and the sites corresponding to the human + 5.8-kb site in chimpanzee, agile gibbon, or Japanese macaque, respectively. The parts in red, green and blue in chimpanzee, agile gibbon, and Japanese macaque, respectively, represent differences from the human sequences. The results are expressed as the average fold of the activity observed for the promoter. The standard deviations indicated are for three independent experiments. The significance of differences was determined by Student’s *t* test at a significance level of p < 0.05 (*) compared to the relative luciferase activity of SN.

To examine whether the sites corresponding to the human + 5.8-kb site in chimpanzee, agile gibbon, or Japanese macaque have potential for trans-activating the *ABO* promoter, we prepared reporter plasmids C(Chi)/SN, C(Gib)/SN, or C(Mac)/SN containing sites corresponding to the human + 5.8-kb site in chimpanzee, agile gibbon, or Japanese macaque, respectively, inserted upstream of the human *ABO* promoter. When these reporters were transiently transfected into K562 cells, the sites corresponding to the human + 5.8-kb site in chimpanzee and agile gibbon enhanced the promoter activity, while that in Japanese macaque did not (Fig. 3). The orthologous site in chimpanzee increased the promoter activity similarly to the human + 5.8-kb site of haplotypes ABOInt1*01, ABOInt1*04 and ABOInt1*06, with an activity higher than that of the site in the agile gibbon. Thus, it was likely that the sites orthologous to the human + 5.8-kb site in chimpanzee and agile gibbon had the potential to enhance the promoter activity, whereas the enhancing activity of the site corresponding to the human + 5.8-kb site in Japanese macaque was deficient.

## Discussion

Here, we compared the genomic sequences of the + 5.8-kb site in human *ABO* with the corresponding sequences in primate species including the chimpanzee, agile gibbon, and Japanese macaque. We found that the sequences of the human + 5.8-kb site and its orthologues including the LTR were conserved among human, chimpanzee, and agile gibbon, whereas in Japanese macaque the site corresponding to the + 5.8-kb site included SINE. In addition, transient transfection experiments using reporter *luciferase* plasmids including the human + 5.8-kb site and its corresponding sites from the three primate species showed that the sequences from chimpanzee and agile gibbon enhanced the transcriptional activity of the *ABO* promoter, whereas that in Japanese macaque did not. These results suggested that the + 5.8-kb site or its orthologues could play a role in *ABO* expression in erythroid lineage cells of human, chimpanzee and agile gibbon. On the other hand, the epithelial cell-specific regulatory region or the + 22.6-kb site appeared to be conserved among primates including human, chimpanzee, gorilla, gibbon, crab-eating macaque, rhesus macaque and marmoset (Fig. 1). Since saliva tests were valid for ABO typing in three primate species, the + 22.6-kb site or its orthologues might be involved in epithelial cell-specific regulation of *ABO* transcription in non-human primates including Japanese macaque, chimpanzee and agile gibbon. Thus, it seemed plausible that the presence of A- and B-antigens on RBCs might be ascribed to emergence of the + 5.8-kb site or its orthologues in intron 1 of *ABO* through genetic evolution in the common ancestral lineage of Hominoidea including human, chimpanzee and agile gibbon.

Almost half of the human genome is derived from transposable elements (TEs)^41^. The majority of TEs consist of retrotransposons including LTRs, SINEs, and long interspersed nuclear elements (LINEs). Retrotransposons are divided according to whether they contain LTRs. Human LTR retrotransposons containing endogenous retroviruses (ERV) and related sequences represent ~ 8% of the human genome whereas non-LTR retrotransposon elements including SINEs and LINEs account for 34%. Most ERV open reading frames have been degraded by mutation. The majority of ERVs are devoid of all coding regions, having undergone recombination between the two flanking LTRs to produce the elements known as solitary LTRs. LTRs including ERV and solitary LTRs may be located at any region of the adjacent genes, including the 5'UTR, intron, exon and 3'UTR. These distributions provide favorable conditions for LTRs regulating the expression of their neighboring genes in different ways, such as promoters, enhancers, selective splicer sites and polyadenylation sites^41–45^. Consistently, the LTR of the + 5.8-kb site and its orthologues are suggested to be involved in the regulation of *ABO* expression in erythroid lineage cells.

Since TEs have repeat sequences spread throughout the genome, and their repeat sequences involve transcription factor binding sites (TFBSs) that act as TE-derived cis-regulatory elements (CREs), it has long been speculated that specific TE families could contribute to dispersion of TFBSs and CREs through the genome, thus facilitating the emergence of a regulatory network controlling a series of cooperative genes^46,47^. It is intriguing to speculate that repeat sequences of TEs play a role in the orchestrated expression of genes encoding glycosyltransferases involved in the production of carbohydrate chains. Indeed, it has been reported that an LTR is a dominant promoter for the gene coding human β1 → 3 galactosyltransferase 5, which is involved in synthesizing the core chain of ABH-antigens^48^. In the first intron of *FUT1*, ENCODE Candidates Cis-regulatory Elements and RepeatMasker indicated distal enhancer-like signatures in the LTRs flanking the SINE, which is assumed to be responsible for H-antigen expression in erythroid lineage cells (Fig. 1A)^19–21^. In addition, it was indicated that the + 5.8-kb site and its orthologues including the LTR are involved in the regulation of *ABO* expression in erythroid lineage cells. Thus, it seems that LTRs could contribute to the *cis*-regulatory network facilitating cooperative expression of the genes involved in the production of carbohydrate chains with A- and B-antigens at the non-reducing end. Future studies will need to investigate the relationship between the LTR at the + 5.8-kb site of *ABO* and the LTRs flanking the SINE in intron 1 of *FUT1* to clarify the mechanism underlying the expression network of genes involved in ABH-antigen production on human RBCs.

## Data Availability

The datasets generated and/or analysed during the current study are available in the Genbank repository, https://ncbi.nlm.nih.gov/nuccore/OP775662 (chimpanzee), https://ncbi.nlm.nih.gov/nuccore/OP775663 (gibbon), https://ncbi.nlm.nih.gov/nuccore/OP775664 (Japanese macaque).
